# CD95/Fas, Non-Apoptotic Signaling Pathways, and Kinases

**DOI:** 10.3389/fimmu.2017.01216

**Published:** 2017-09-27

**Authors:** Matthieu Le Gallo, Amanda Poissonnier, Patrick Blanco, Patrick Legembre

**Affiliations:** ^1^Centre Eugène Marquis, Rennes, France; ^2^Equipe Labellisée Ligue Contre Le Cancer, INSERM U1242 COSS Institut National de la Santé et de la Recherche Médical, Rennes, France; ^3^Université de Rennes-1, Rennes, France; ^4^Centre Hospitalier Universitaire (CHU) de Bordeaux, Université de Bordeaux, Bordeaux, France; ^5^UMR CNRS 5164, Bordeaux, France

**Keywords:** apoptosis, endothelium, FasL, Fas, lupus metastasis, migration, tyrosine kinase

## Abstract

Endothelial cells lining new blood vessels that develop during inflammatory disorders or cancers act as doors that either allow or block access to the tumor or inflamed organ. Recent data show that these endothelial cells in cancer tissues and inflamed tissues of lupus patients overexpress CD95L, the biological role of which is a subject of debate. The receptor CD95 (also named Fas or apoptosis antigen 1) belongs to the tumor necrosis factor (TNF) receptor superfamily. Its cognate ligand, CD95L, is implicated in immune homeostasis and immune surveillance. Because mutations of this receptor or its ligand lead to autoimmune disorders such as systemic lupus erythematosus (SLE) and cancers, CD95 and CD95L were initially thought to play a role in immune homeostasis and tumor elimination *via* apoptotic signaling pathways. However, recent data reveal that CD95 also evokes non-apoptotic signals, promotes inflammation, and contributes to carcinogenesis; therefore, it is difficult to dissect its apoptotic effects from its non-apoptotic effects during pathogenesis of disease. CD95L is cleaved by metalloproteases and so exists in two different forms: a transmembrane form and a soluble ligand (s-CD95L). We recently observed that the soluble ligand is overexpressed in serum from patients with triple-negative breast cancer or SLE, in whom it contributes to disease severity by activating non-apoptotic signaling pathways and promoting either metastatic dissemination or accumulation of certain T cell subsets in damaged organs. Here, we discuss the roles of CD95 in modulating immune functions *via* induction of mainly non-apoptotic signaling pathways.

## Introduction

Accumulating evidence suggests that high endothelial venule (HEV) density in the tumor stroma is a strong predictor of infiltration by CD4^+^ T, CD8^+^ T, and B cells. Although this statement seems obvious because the blood vessels carry oxygen, nutrients, and cells to the organs, selective infiltration by certain T cell subsets at the expense of others is a very complex process. Here, we describe recent data suggesting new roles for CD95L in this process and, more generally, new biological roles for CD95L in progression of cancer and autoimmune disorders such as systemic lupus erythematosus (SLE).

The immune system comprises specialized cells that protect the body from infection and cell transformation. Antigen (Ag)-presenting cells (APCs) survey tissues for non-self Ags and capture pathogenic agents. Ags are processed and presented at the cell surface in the context of major histocompatibility complex (MHC) molecules. Similar to macrophages and B lymphocytes, dendritic cells are professional APCs. APCs present Ags on MHC class II molecules and not only participate in activating non-self-Ag-recognizing T cells but also contribute to elimination of T-cells that recognize self-Ags with a too strong affinity; the latter process is called peripheral tolerance. αβ T cells display either CD4 or CD8 on their surface. CD4^+^ T helper (Th) lymphocytes bind Ag presented in the context of MHC class II molecules expressed by professional APCs and produce cytokines that drive the immune response. CD8^+^ T-lymphocytes recognize antigenic peptides presented by MHC class I molecules expressed on the surface of all cells (apart from some transformed or infected cells), resulting in elimination of these target cells.

The long list of Th subset lineages shows the complexity of the mechanisms developed by the immune system to respond to the diversity of infectious agents and cancers. Polarization of CD4^+^ T-cell subsets occurs *via* exposure to cytokines, microbiota, high salt concentrations, or high-fat diet (1) that induce expression of different transcription factors such as STAT1, STAT4, and T-bet (for Th1 cells), STAT6 and GATA-3 (for Th2 cells), FoxP3 (for regulatory CD4^+^ T cells; Tregs), Bcl6 (for follicular Th cells; TFH) (2), RORγt, RORα, and STAT3 (for Th17 cells), T-bet and AhR (for Th22 cells) (3), and PU-1 for Th9 cells (4). These T cells express a set of cytokines that organize the entire immune system (innate and adaptive immune cells), so that it responds adequately (or not) to infection and transformation. CD8^+^ cytotoxic T-lymphocytes destroy target cells and spare bystanders. Recent studies show that the immune landscape (intensity and distribution) within tumors may serve as a prognostic marker for the risk of disease progression and help to predict the efficiency of chemotherapeutic/radiotherapeutic/immunotherapeutic regimens (5).

**Figure 1:**
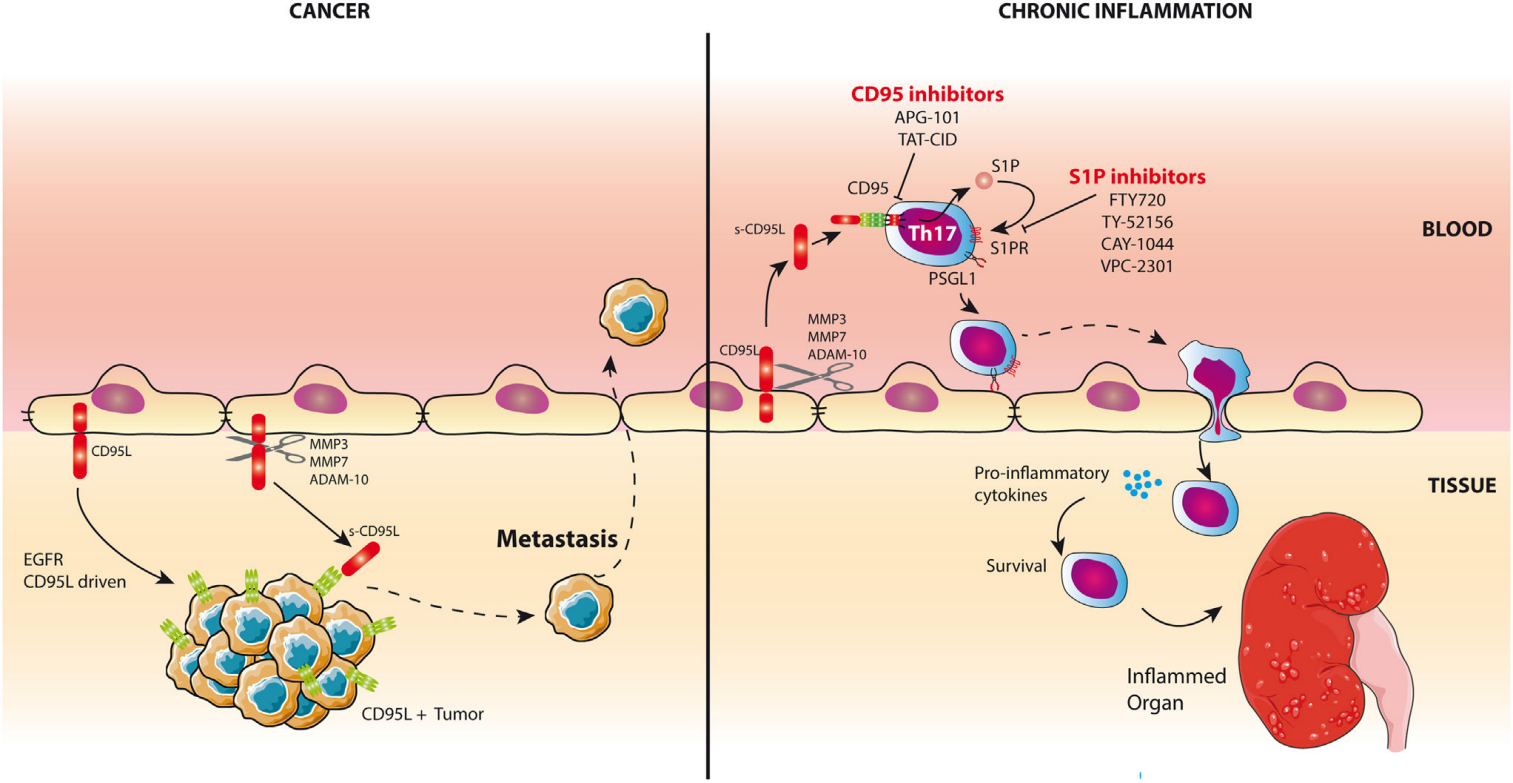
CD95L is a chemoattractant for inflammatory T cells and triple-negative breast cancer (TNBC) cells. *Left panel*: CD95L is expressed by endothelial cells in damaged organs in lupus patients and in TNBCs. Cleavage of CD95L by metalloproteases releases a soluble form of the ligand. In TNBCs, s-CD95L acts as a chemottractant for tumor cells promoting metastasis. *Right panel*: In chronic inflammatory pathologies like systemic lupus erythematosus, s-CD95L enhances the trafficking of Th17 T cells in damaged organs through the activation of sphingosine 1 phosphate (S1P) pathway. Infiltrating Th17 cells release pro-inflammatory cytokines. Th17 recruitment can be blocked by S1P or CD95 inhibitors.

To simplify, APCs present Ags to CD4^+^ Th cells, which in turn orchestrate activation of the immune response *via* a cellular (Th1/Th17) or humoral (Th2) response, or by downregulating the intensity/kinetics of the adaptive immune response by promoting differentiation of Tregs. CD4^+^ Th subsets regulate the adaptive immune response by finely tuning the response to particular infectious agents and cancer cells. In addition, the efficiency of the immune response relies on the ability of these cells to cross blood vessels and migrate within inflamed or transformed tissue to reach the target. Selectins, integrins, and chemokine receptors play a central role in T cell extravasation, which involves several different steps: arrest of cells on the endothelial surface, adhesion and spread, crawling, and transendothelial migration (Figure 1). Once immune function has been achieved, effector T cells are eliminated by molecular mechanisms based on death receptors (DRs) or the BH3-only factor BIM (according to the affinity of the immune cell for the stimulating Ag, i.e., low versus high affinity) (6); however, some Ag-specific memory T cells persist.

Because TCR activation induces *de novo* expression of CD95L by CD8^+^ T-cells, this ligand was regarded as a lethal weapon used by these cells to eliminate target cells (7–9). However, novel studies revisited the biological role of CD95 and found that this receptor also activates non-apoptotic signaling pathways, thereby enhancing pro-inflammatory functions that contribute to the severity of autoimmune disorders and cancers. For instance, CD95/CD95L participates in selective accumulation of pro-inflammatory Th17 cells in the damaged organs of lupus patients (10).

## DRs Belonging to the Tumor Necrosis Factor (TNF) Receptor (TNF-R) Family

### CD95

Death receptors including CD95 (11), TNFR1 (12), DR4 (13), DR5 (14), and DR6 (15) belong to the TNF-R superfamily. These type I transmembrane proteins share common features, including extracellular amino-terminal cysteine-rich domains (16, 17) that contribute to both ligand specificity (18) and receptor preassociation (19–21) and the death domain (DD) that encompasses an 80 amino acid sequence located in the cytoplasmic tail; this domain transmits the apoptotic signal through protein–protein interactions (PPIs) (22, 23). Among them, adaptor proteins that interact with the DD, TRADD, and FADD are instrumental in implementing cell death pathways (24–27). It is noteworthy that TNF-R members do not possess enzymatic activity rather they rely on dynamic PPI formation to recruit enzymes and signals. This property accounts for the versatility and pleiotropy of these receptors in terms of triggered signaling pathways.

Based on the fact that DRs were originally identified and cloned according to their ability to elicit apoptosis, some of these receptors (mainly CD95 for historical reasons) are still considered by a part of the scientific community to be monomaniac death inducers, even though this is clearly not the case (28–31). Historically, a monoclonal antibody designated “apoptosis antigen 1” was selected for its ability to trigger cell death in tumor cells (32); this antibody recognizes Fas/CD95 (11). Accordingly, most future studies investigated the role of the CD95-mediated apoptotic signaling pathway during progression of cancers and inflammatory disorders. Nonetheless, and unlike TNFα-targeting therapeutics, all molecules acting as CD95 agonists failed to reach clinical trials. This is probably due to the fact that CD95 does not only function as a DR and that inhibiting it rather than activating it may be a more attractive therapeutic option for patients suffering from cancers and chronic inflammatory disorders. Although the molecular events that generate the apoptotic signal induced by CD95 are well defined, it is not clear how the receptor activates non-apoptotic signaling pathways (i.e., NF-κB, MAPK, and PI3K).

### CD95L

Transmembrane CD95L (m-CD95L) is a potent inducer of cell death (33). m-CD95L can be cleaved by different metalloproteases and although s-CD95L interacts with CD95 it fails to trigger cell death (34, 35). It does, however, trigger non-apoptotic signaling pathways (36, 37). Until now, no-one has explained how the interaction between a unique ligand (CD95L) and a unique receptor (CD95) induces such different signaling pathways. It may be that differences in the kinetics of CD95 aggregation, or its internalization and/or membrane distribution, may account for the induction of one cue at the expense of another. Our hypothesis is that the magnitude and pace of execution of the above mechanisms may depend on the stoichiometry of the ligand interacting with CD95. Indeed, while s-CD95L exists mainly as a homotrimer (35), its membrane-bound counterpart exists as multi-aggregated homotrimers (38). This is an important feature of TNF family members; these membrane-bound ligands are processed by metalloproteases to release a soluble ligand counterpart (30). The difference in their stoichiometry (soluble and homotrimeric ligand versus membrane-bound and multi-aggregated homotrimeric CD95L) and/or their conformational effect on the receptor (natural ligand versus agonistic antibodies) might explain the differences in activated cell signaling pathways (39, 40). Similarly, TNFα can be cleaved by the metalloprotease TACE (41, 42) to release a soluble form of the cytokine (s-TNFα). Whereas s-TNFα only activates TNFR1, m-TNFα binds and activates both TNFR1 and TNFR2 (43). Activating TNFR1 induces cellular processes ranging from cell death (apoptosis or necroptosis) to cell proliferation, migration, and differentiation; implementation of these cellular responses reflects the dynamic formation of PPIs following receptor activation (44).

The transmembrane form of CD95L was originally detected at the surface of activated lymphocytes (45) and natural killer (NK) cells (46), where it not only contributes to eliminating tumor and infected cells but also kills T-cells through a process called activation-induced cell death (AICD) and dendritic cells (47). CD95L is also expressed on the surface of neurons (48), corneal epithelia, and endothelia (49, 50), where it prevents infiltration of immune cells and thereby inhibits the spread of inflammation within these sensitive organs, which are commonly referred to as “immune-privileged” sites. Of the different types of intestinal epithelial cells, Paneth cells express CD95L (51), although no expression has been reported in normal villus epithelium. However, mouse villus epithelial cells express CD95L after exposure to oxidative stress, and this expression may affect the way the immune response reacts to the microbiota (52).

More recently, different groups have shown that, even if CD95L can be detected on the surface of immune cells, the ligand is mainly found on the surface of endothelial cells lining blood vessels feeding cancer tissues (37, 53) or damaged organs in lupus patients (10, 36). Although many studies have examined expression of CD95L, few report expression of this ligand on the surface of endothelial cells, probably because they used the incorrect antibodies. Indeed, although several anti-CD95L antibodies exist, only one (clone G247-4) gives reliable results in immunohistochemistry experiments (54). In patients with inflammatory bowel diseases, G247-4 detected CD95L in HEVs expressing HEV markers such as MAdCAM-1 and MECA-79 (55). Strikingly, CD95L-expressing HEVs observed in cow’s-milk-sensitive enteropathy (CMSE) and celiac disease were surrounded by lymphoid follicles. Moreover, overexpression of m-CD95L in CMSE correlated with increased concentrations of serum CD95L, enlargement of mucosal lymphoid nodules in the gastrointestinal tract (GIT), and infiltration by intraepithelial lymphocytes (56). These data suggest that, after cleavage by metalloproteases, s-CD95L attracts leukocytes to the inflamed GIT.

We recently detected CD95L in endothelial cells lining blood vessels in inflamed skin of lupus patients and in tumor tissues from women with triple-negative breast cancer (TNBC) (10, 37). This ligand is cleaved by metalloprotease to yield s-CD95L, which is present in the serum of these patients at higher amounts than in that of healthy donors. Our initial study showed that the naturally processed ligand triggers PI3K and Ca^2+^ signaling pathways in activated T cells, which then accumulate in the inflamed organs of SLE patients (36); this raises the question of whether all T cells, or only certain Th subsets, respond to s-CD95L and contribute to SLE pathology by accumulating in inflamed organs. As aforementioned, TCR engagement leads to the overexpression of CD95 (57) and CD95L (9) rendering activated T-cells susceptible to apoptosis and contributing to their elimination by AICD (58, 59). Overexpression of m-CD95L in activated CD4^+^CD25^high^Foxp3^+^ Tregs serves to eliminate CD8^+^ T-cells (60). Strikingly, blood Tregs isolated from patients affected by head and neck squamous cell carcinoma and stimulated with anti-CD3 mAb and IL-2 exhibit a higher level of CD95L as compared to that present at the surface of Tregs from healthy donors, providing a potential explanation for the elimination of tumor-infiltrating CD8^+^ effector T-cells in these cancer patients (60). Similar to Tregs, myeloid-derived suppressor cells (MDSCs) inhibit adaptive and innate immunity and accumulate in the blood of cancer patients, chronic inflammation and infection. They consist of two major subsets, granulocytic (G-MDSC) and monocytic (M-MDSC) subsets, producing immune suppressive factors including arginase I, inducible nitric oxide synthase, and cytokines and thereby, inhibiting effector T-cell functions (61). Similar to Tregs, MDSCs express CD95 and die in response to CD95L stimulation (62). Consequently, *gld* BALB/c mice, which express a mutated CD95L unable to bind its receptor, show an accumulation of MDSCs as compared to their wild-type counterpart (62) indicating that CD95/CD95L pair is instrumental in the homeostasis of this immunosuppressive cell population. Interestingly, a syngeneic graft of Lewis lung carcinoma in CD95-KO mice (C57BL/6 strain) did not reveal any increase in the number of MDSCs in the spleen or the tumor tissues as compared to wild-type mice (63). The G-MDSC subset is the prevalent population of MDSCs in different tumor models with an expected ratio of 60% of G-MDSCs (63). An in-depth analysis of LCC-infiltrating MDSCs highlighted that the loss of CD95L skewed cell populations toward the M-MDSC subset, which displayed a higher immunosuppressive activity as compared to G-MDSCs (63). Overall, these findings indicate that the loss of CD95L or CD95 might create an immunosuppressive environment by increasing the number of Tregs and MDSCs and/or by increasing in the M-MDSC/G-MDSC ratio and thereby renders mice more susceptible to tumor progression as compared to the wild-type mice.

Th17 cells contribute to kidney damage and pathology severity in SLE patients (64, 65). Although blood Tregs express higher CD95 levels than Th17 cells, this former regulatory T-cell did not respond to s-CD95L (10). On the other hand, Th17 cells underwent endothelial transmigration and accumulated in inflamed organs of lupus patients in a CD95-dependent manner. In an effort to explain the CD95-driven accumulation of Th17 cells in damaged organs, we showed that these Th17 cells exposed to s-CD95L upregulate expression of the adhesion molecule PSGL-1, which not only promotes tethering of lymphocytes to endothelial cells and subsequent rolling (Figure 1) but also provokes secretion of effector cytokines when highly expressed by T cells (66). Indeed, Bradley team suggests that PSGL-1 expression affects the interaction of T-cells with APCs and more specifically, among the CD4^+^ Th1, activation of cells with the highest PSGL-1 levels are the most proliferative effectors with the greatest capacity for effector cytokine secretion (66) probably through a PI3K-driven mechanism (67). Therefore, s-CD95L might fuel the inflammatory process in lupus patients first by mediating accumulation of Th17 cells in organs and second by altering the pattern of cytokines released in the inflamed tissues (Figure 1). The sphingosine 1 phosphate (S1P) signaling pathway is a critical regulator of many pathophysiological processes (68). Unlike Tregs, exposure of Th17 cells to s-CD95L causes them to upregulate expression of S1P (10). Activating the S1P signaling pathway in Th17 cells exposed to s-CD95L provides a molecular mechanism by which Th17 cells transmigrate in the presence of this soluble ligand. Successful development of the S1P analog FTY720 and its use as a drug to treat multiple sclerosis (69) shows that it is possible to target S1P signaling in humans and, therefore, that S1P antagonists would be an attractive therapeutic option for preventing CD95-mediated transmigration of Th17 cells in SLE patients (Figure 1). Interestingly, the ectopic expression of CD95L by endothelial cells also promotes transmigration of CD95-expressing myeloid cells by tethering cells and slowing cell rolling (70).

Nonetheless, the role of endothelial CD95L remains controversial because, while Coukos et al. observed that the membrane-bound ligand served as a barrier to prevent CD8 T-cell extravasation (while sparing Treg accumulation in the tumor tissue) (53) (Figure 1), we found that CD95L on the surface of endothelial cells can be cleaved by metalloproteases to create a gradient that is responsible for accumulation of Th17 cells in inflamed organs (extravasation) (10, 36) or the metastatic dissemination of TNBC cells (intravasation) (37) (Figure 1). In agreement with our data, initial experiments with CD95L-expressing tumor cells did not promote carcinogenesis by killing tumor-infiltrating immune cells but instead caused acceleration of tumor rejection (when compared with CD95L-negative tumor cells), which was associated with massive recruitment of neutrophils to the transplanted area (71). Moreover, CD95L-expressing cells provide T cells with a survival signal (72). For instance, CD95L-expressing β-islets increase CD8^+^ activation, leading to organ damage; also, the same experiment in NOD mice backcrossed with MRL^lpr/lpr^ mice (exhibiting loss of CD95) failed to stimulate T cells, meaning that the mice did not develop diabetes (73). In summary, accumulating evidence suggests that CD95L acts as a direct chemoattractant for neutrophils, macrophages (71, 74–76), T lymphocytes (36), and malignant cells in which the CD95-mediated apoptotic signal is non-productive (77, 78). This ligand can also exert an indirect chemoattractant effect by promoting the expression of IL-8 and MCP-1 recruiting phagocytes (79, 80). Recent data highlight that inflammatory Th17 cells fail to differentiate when CD95 expression is downregulated (81), suggesting that experiments with CD95L-expressing tumor cells may promote tumor graft rejection by inducing a Th17 differentiation-driven inflammatory environment. The cytokine microenvironment also plays a pivotal role in the CD95 signaling pathway, as highlighted by the fact that addition of TGF-β to the microenvironment of CD95L-expressing tumor cells inhibits their rejection (71). This conclusion remains difficult to reconcile with the fact that TGF-β is a crucial cytokine for Th17 cell development and maintenance (82). Nonetheless, TGF-β1 is also instrumental in Treg differentiation and thereby prevents autoimmunity (83). The current consensus is that TGF-β orchestrates the differentiation of Th17 and Treg subsets by promoting the expression of both Foxp3 and RORγt (84); but, TGF-β treatment leads exclusively to Treg differentiation, because Foxp3 inhibits RORγt transcriptional activation (84). On the other hand, the presence of IL-6 and/or IL-21 (85) abrogates this inhibition and favors the Th17 differentiation (86, 87). In addition, recent data highlight that, in the presence of TGF-β, differentiated Th17 cells can express regulatory cytokines including IL-9 and IL-10 whereas those derived from medium devoid of TGF-β possess a more inflammatory signature and express Th1-associated molecules such as IFN-γ (88). Therefore, TGF-β may favor differentiation of Tregs or “regulatory” Th17 cells and prevent elimination of CD95L-expressing tumor cells.

Some years ago, we were surprised to observe that there was no correlation between the magnitude of the CD95-mediated non-apoptotic signaling pathways and resistance to cell death, thereby ruling out the hypothesis that the CD95-mediated non-apoptotic signaling pathway counteracts the apoptotic pathway (89). Recent data show that the simultaneous induction of apoptotic and non-apoptotic signaling pathways in cells exposed to cytotoxic CD95L is necessary for an efficient immune response because CD95-mediated non-apoptotic signaling pathways in dying cells allow production of cytokines (including MCP-1 and IL-8) that in turn recruit professional phagocytes *via* a cellular inhibitor of apoptosis (cIAP)-1 and a cIAP-2-driven molecular mechanism (80). This original and cooperative interplay between apoptotic and non-apoptotic signaling pathways promotes accumulation of macrophages and phagocytes, which then engulf dying cells. These results not only explain the rapid elimination of apoptotic cells *in vivo* but also may provide a molecular link between cell death and the manner in which Ags (derived from transformed or infected cells) are presented to mount an efficient or impaired immune response.

Of note, s-CD95L can also fuel carcinogenesis by promoting angiogenesis (90, 91), probably through the stimulation of vascular endothelial growth factor (VEGF) secretion by endothelial cells (91). Because in cancer tissues, VEGF, IL-10, and prostaglandin E2 cooperatively stimulate the expression of m-CD95L by endothelial cells (53), we envision that the presence of a metalloprotease, which remains to be identified, can release s-CD95L and thereby triggers a positive feedback loop accelerating carcinogenesis.

## Two CD95L and Many Cell Signaling Pathways

CD95L is a transmembrane cytokine whose ectodomain can be cleaved close to the plasma membrane by metalloproteases such as MMP3 (92), MMP7 (93), MMP9 (94), and ADAM10 (95, 96); CD95L is then released into the bloodstream as s-CD95L. Metalloprotease-mediated cleavage of CD95L releases a homotrimeric ligand that does not trigger the apoptotic program (35). Indeed, experiments show that hexameric CD95L represents the minimal level of self-association required to signal apoptosis (39). The homotrimeric ligand has long been considered an inert molecule that only competes with its membrane-bound counterpart to antagonize the death signal (34, 35). However, recent work shows that this metalloprotease-cleaved CD95L actively aggravates inflammation and autoimmunity in patients with SLE by inducing non-apoptotic signaling pathways, including the NF-κB and PI3K pathways (36, 97).

It is noteworthy that there are different forms of s-CD95L. Although the soluble form of CD95L generated by MMP7-driven cleavage of its ^113^ELR^115^ sequence (the CD95L stalk region) induces apoptosis (93), its counterpart (cleaved between serine 126 and leucine 127) does not (35, 36, 97). For instance, s-CD95L in the bronchoalveolar lavage (BAL) fluid of patients suffering from acute respiratory distress syndrome (ARDS) is oxidized at methionines 224 and 225, thereby promoting aggregation of the soluble ligand and boosting its cytotoxic activity (98). The stalk region of CD95L, corresponding to amino acids 103–136, contains all metalloprotease cleavage sites described in the literature. Surprisingly, this region is conserved in s-CD95L detected in ARDS patients, participates in CD95L multimerization, and accounts for ligand-mediated cytotoxicity and damage to the lung epithelium (98). Of note, in ARDS BAL fluid, additional oxidation of s-CD95L occurs at methionine 121, thereby preventing cleavage of CD95L by MMP7 and potentially explaining why this cytotoxic ligand retains its stalk region and contributes to disease pathology (98). Nonetheless, preservation of this region raises the question of whether an as-yet-unidentified MMP7-independent cleavage site exists in the juxtamembrane region of CD95L, or whether the ligand detected in ARDS patients corresponds in fact to the full-length form of CD95L embedded in exosomes (99, 100). Indeed, exosome-bound CD95L can be expressed by human prostate cancer cells (i.e., the LNCaP cell line) and evokes apoptosis in activated T lymphocytes (101). In addition to MMPs, plasmin can also cleave the m-CD95L expressed by neo-vessels in cancers (102). Plasminogen activator inhibitor-1 (PAI-1) inhibits the activity of urokinase-type plasminogen activator, which converts plasminogen into its active form, plasmin. PAI-1 is a good prognostic marker in patients affected by glioblastoma or breast and colon cancers (102). Plasmin cleaves CD95L between the amino acid residues Arg144 and Lys145 within its trimerization domain and releases a soluble ligand, which surprisingly triggers cell death in endothelial cells (102). Because no analysis of the s-CD95L stoichiometry has been realized in this study, it is difficult to decipher whether the soluble plasmin-generated CD95L corresponds to an aggregate of homotrimers or whether cleavage by plasmin releases a peculiar homotrimeric ligand, which interacts with other components accounting for its unexpected apoptotic effect.

Overall, these findings emphasize the importance of finely characterizing the stoichiometry of naturally processed CD95L in the serum of patients affected by cancers or chronic/acute inflammatory disorders to predict the biological role (i.e., apoptotic versus non-apoptotic) of this soluble ligand. Such investigations will improve our understanding of the molecular mechanisms set in motion by this ligand, and our appreciation of its downstream biological functions.

## Interplay Between Caspase-8 and Kinases in the CD95 Signaling Pathways

As mentioned earlier, CD95 does not possess any enzymatic activity; however, by triggering a large number of PPIs, it activates different signaling pathways resulting in different clinical outcomes. Here, the aim is not to exhaustively describe the initial events of the CD95-mediated signaling pathway, but rather to focus on the role of kinases in these pathways. Unlike m-CD95L, whose interaction with CD95 leads to formation of the apoptotic complex *Death-Inducing Signaling Complex* (DISC), s-CD95L leads to formation of a molecular complex devoid of FADD and caspase-8, which instead recruits and activates the Src kinase c-yes *via* production of NADPH oxidase 3 and ROS (36, 78); this unconventional receptosome is designated the motility-inducing signaling complex (MISC) (36, 37). This process was also observed in apoptotic-resistant glioblastoma cells, in which CD95 stimulation triggers recruitment of a molecular complex that contains the Src tyrosine kinase c-yes (78). This complex promotes micrometastatic dissemination of glioblastoma cells. Similarly, upon injury to the central nervous system, neutrophils and macrophages overexpress CD95L, which promotes migration of these cells *via* activation of the src kinase Syk (76). Of note, while deleting CD95 from neural cells has no effect on pathology progression, deleting CD95L from myeloid cells accelerates spinal cord recovery by reducing the infiltration of neutrophils and macrophages and the magnitude of inflammation. These findings clearly show that the CD95-mediated apoptotic response is not responsible for CD95/CD95L function in this pathological context, but that the ligand/receptor pair is required for accumulation of inflammatory cells contributing to the degradation of the organ.

**Figure 2:**
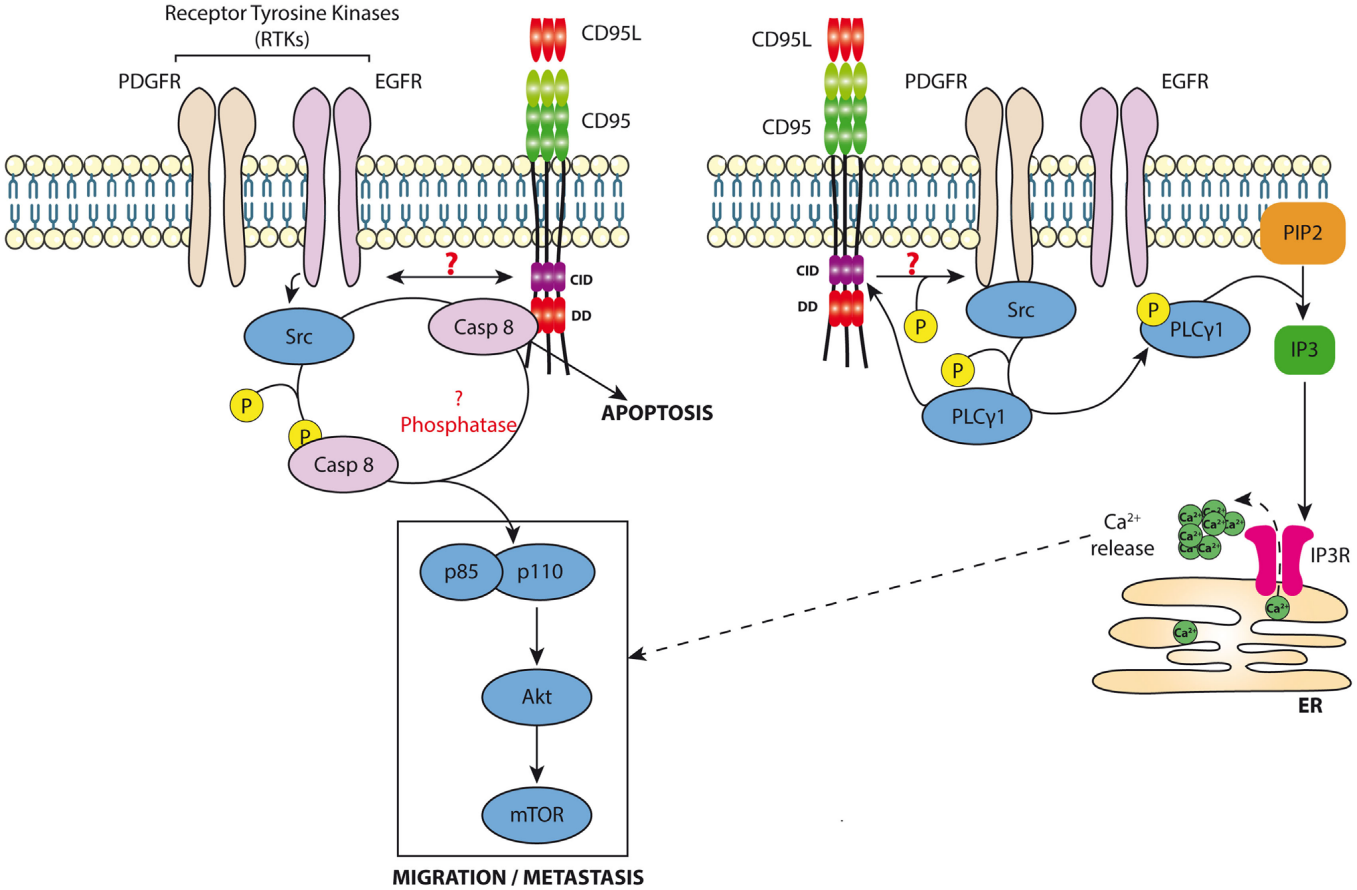
Non-apoptotic signaling pathway induced by CD95. *Left panel*: Cancer cells exposed to s-CD95L induce the recruitment of RTKs (i.e., EGFR or PDGFR) by CD95 leading to the binding and activation of src. In turn, src kinases can phosphorylate caspase-8 neutralizing its enzymatic activity and promoting its role in the activation of the PI3K signaling pathway (i.e., recruitment of p85) and cell migration. *Right panel*: CD95 favors RTK activation leading to Src recruitment. s-CD95L interaction with CD95 induces binding of PLCγ1, which is activated through a src-dependent phosphorylation. Activated PLCγ1 hydrolyzes phosphatidylinositol 4,5-bisphosphate (PIP2), releasing inositol triphosphate (IP3), which activates IP3Rs on endoplasmic reticulum promoting calcium release into the cytoplasm that contributes to the CD95-mediated cell migration signaling pathway.

Although our data showed no trace of caspase-8 in the MISC of TNBC cells exposed to s-CD95L (37), we cannot rule out the presence of trivial amounts of this protease in MISC (undetectable by western blotting). Caspase-8 is known to participate in cell migration mainly through an enzymatic-independent mechanism. Src kinases abrogate the protease activity of caspase-8 by phosphorylating it at tyrosine 380 (Y380) (103); this favors recruitment of the PI3K adaptor p85 alpha subunit, thereby activating the PI3K signaling pathway (104) (Figure 2). A study revealed that in hepatocytes, hyperosmolarity induces association between a receptor tyrosine kinase (RTK) [called epidermal growth factor receptor (EGFR)] and CD95 through a JNK and PKC-dependent mechanism that remains to be elucidated (105). Strikingly, this study showed that CD95/EGFR increases both DISC formation and the caspase-8-dependent apoptotic response in hepatocytes. More recent data show that EGFR phosphorylates caspase-8 at Y380 through activation of Src kinases, and that this RTK-mediated translational modification prevents triggering of the CD95-mediated apoptotic signaling pathway (103). Several reports confirm that phosphorylation of caspase-8 at Y380 inhibits its protease activity (106, 107). The death effector domains of caspase-8 are both sufficient and necessary for EGFR-dependent cell migration (108). Because caspase-8 phosphorylation at Y380 recruits Src kinases, including Fyn, Lyn, and Src kinases, it is tempting to speculate that this process constitutes a positive feedback loop, which in turn phosphorylates additional caspase-8 molecules (107) (Figure 2). We recently observed that exposure of TNBC cells overexpressing EGFR to s-CD95L induces association of CD95 with EGFR, thereby promoting cell migration and metastatic dissemination of these cancer cells (37). RTK-driven Y380 phosphorylation of caspase-8 might prime certain cancer cells to become unresponsive to the apoptotic signal triggered by cytotoxic CD95L, thereby diverting this cell signaling pathway toward promoting cell migration, an essential step in cancer cell metastasis (Figure 2). These data are in agreement with those published in a recent study showing that another RTK, PDGFR-β, is recruited by CD95 in colon cancer cells exposed to CD95L to trigger cancer cell metastasis (109). CD95 triggers phosphorylation of PDGFR at tyrosine 1021, thereby promoting recruitment of phospholipase Cγ1 (PLCγ1) (109). Of note, we recently showed that PLCγ1 directly docks onto the calcium-inducing domain of CD95 (10), suggesting that PDGFR-β-dependent recruitment of PLCγ1 might increase the amount of lipase recruited by CD95, and/or sustain its presence at the plasma membrane to increase production of inositol trisphosphate, which activates the Ca^2+^ response and diacylglycerol to stimulate conventional and novel PKCs (Figure 2). CD95 induces T cell migration by activating PLCγ1 (36), which necessitates its Src kinase-driven phosphorylation at tyrosine 783 (Y783) (110). Also, the RTK called hepatocyte growth factor receptor or c-Met binds and sequesters CD95 to impair its interaction with CD95L, highlighting another molecular mechanism used by RTKs to prevent apoptosis (111).

These findings show that CD95-mediated recruitment of RTKs, or *vice versa*, may correspond to a physiological process that simultaneously inhibits the apoptotic signal by phosphorylating caspase-8, thereby promoting cell motility by activating the PI3K signaling pathway. However, it is unclear how CD95 interacts with RTKs.

## Conclusion and Perspectives

Although the CD95/CD95L interaction can eliminate malignant cells by promoting formation of the DISC, it can also promote carcinogenesis by maintaining inflammation and/or by inducing metastatic dissemination (36, 76–78, 97, 112, 113). The molecular mechanisms underlying the switch between these different signaling pathways remain enigmatic. An important question to be addressed is how the magnitude of CD95 aggregation regulates formation of “Death”- versus “Motility”-ISCs. Answering these questions will lead to development of new therapeutic agents with the ability to prevent the spread and chronicity of inflammation, or to impinge on carcinogenesis, at least with respect to pathologies associated with increased s-CD95L, such as cancers [e.g., pancreatic cancer (114), large granular lymphocytic leukemia, breast cancer (37), and NK cell lymphoma (115)] and autoimmune disorders [e.g., rheumatoid arthritis and osteoarthritis (116), and graft-versus-host-disease (117, 118), or SLE (36, 119)].

This review highlights that CD95L is frequently detected overexpressed at the surface of endothelial cells covering blood vessels in tissues of patients affected by inflammatory disorders or cancers. Although its role remains to be elucidated, growing evidence suggest that this ectopic expression can be a biological marker whose combination with a “lethal” metalloprotease might turn out to enhance the severity of autoimmune disorders and cancers. An extensive review of the CD95/CD95L role in the function of endothelial cells indicates that besides its role in the immune system, CD95 can directly regulate angiogenesis either by implementing cell death when encountering its transmembrane ligand or by promoting survival and migration of these endothelial cells when exposed to s-CD95L. Finally, CD95 can also stimulate the production of nitric oxide and thereby control blood pressure (120) revealing its complex biological role in the context of the endothelium survival, proliferation, and function.

## Author Contributions

All authors listed have made a substantial, direct, and intellectual contribution to the work and approved it for publication.

## Conflict of Interest Statement

The authors declare that the research was conducted in the absence of any commercial or financial relationships that could be construed as a potential conflict of interest.
